# A comparison of the surgical mortality due to colorectal perforation at different hospitals with data from 10,090 cases in the Japanese National Clinical Database

**DOI:** 10.1097/MD.0000000000005818

**Published:** 2017-01-13

**Authors:** Takeshi Ohki, Masakazu Yamamoto, Hiroaki Miyata, Yasuto Sato, Yoshihisa Saida, Tsuyoshi Morimoto, Hiroyuki Konno, Yasuyuki Seto, Koichi Hirata

**Affiliations:** a Department of Surgery, Institute of Gastroenterology, Tokyo Women's Medical University; b National Clinical Database; c Department of Health Policy and Management, School of Medicine, Keio University; d Department of Public Health, Tokyo Women's Medical University; e Department of Surgery, Toho University Ohashi Medical Centre, Tokyo; f Department of Radiology, St. Marianna University School of Medicine, Kawasaki; g The Japanese Society of Gastroenterological Surgery, Tokyo; h Second Department of Surgery, Hamamatsu University School of Medicine, Hamamatsu; i Department of Gastrointestinal Surgery, Graduate School of Medicine and Faculty of Medicine, The University of Tokyo; j The Japanese Society for Abdominal Emergency Medicine, Tokyo; k First department of surgery of Sapporo Medical university school of Medicine, Sapporo; l Department of Surgery, JR Sapporo Hospital, Sapporo, Japan.

**Keywords:** colorectal perforation, differences in hospitals, fatality rate, National Clinical Database (NCD), observed-to expected mortality ratio (O/E ratio)

## Abstract

Colorectal perforation has a high rate of mortality. We compared the incidence and fatality rates of colorectal perforation among different hospitals in Japan using data from the nationwide surgical database.

Patients were registered in the National Clinical Database (NCD) between January 1st, 2011 and December 31st, 2013. Patients with colorectal perforation were identified from surgery records by examining if acute diffuse peritonitis (ADP) and diseases associated with a high probability of colorectal perforation were noted. The primary outcome measures included the 30-day postsurgery mortality and surgical mortality of colorectal perforation. We analyzed differences in the observed-to-expected mortality (O/E) ratio between the two groups of hospitals, that is, specialized and non-specialized, using the logistic regression analysis forward selection method.

There were 10,090 cases of disease-induced colorectal perforation during the study period. The annual average postoperative fatality rate was 11.36%. There were 3884 patients in the specialized hospital group and 6206 in the non-specialized hospital group. The O/E ratio (0.9106) was significantly lower in the specialized hospital group than in the non-specialized hospital group (1.0704). The experience level of hospitals in treating cases of colorectal perforation negatively correlated with the O/E ratio.

We conducted the first study investigating differences among hospitals with respect to their fatality rate of colorectal perforation on the basis of data from a nationwide database. Our data suggest that patients with colorectal perforation should choose to be treated at a specialized hospital or a hospital that treats five or more cases of colorectal perforation per year. The results of this study indicate that specialized hospitals may provide higher quality medical care, which in turn proves that government policy on healthcare is effective at improving the medical system in Japan.

## 1 Introduction

Colorectal perforation has a high fatality rate ranging from 8.5% to 33.3%.^[1–6]^ Therefore, it should be diagnosed and appropriately treated as early as possible, as most patients require surgery. In addition, sharing information among the medical staff, such as the treatment outcome, surgical procedures, and incidence rate, is very important. Thus far, only isolated data collected from small numbers of patients have been available to determine the mortality of colorectal perforation.

The National Clinical Database (NCD), a new Japanese surgical registration system, was recently developed. This system allows surgeons to register patients’ data through a website. The NCD is a nationwide project similar to the United States’ surgical assessment system developed by the American College of Surgeons—National Surgical Quality Improvement Program.^[7]^ The NCD was established in April 2010 and registration began in 2011. More than 4000 facilities were registered in the first 3 years. In association with the certification board of the Japan Surgical Association,^[8]^ more than 4,000,000 surgical cases have been collected and entered into the database. We believe that all the information related to colorectal perforation in Japan since 2010 is available in NCD. However, the data are not well organized, and no specific category exists for colorectal perforation. Thus, it is vital to extract the relevant data on colorectal perforation and organize it into a more specific category for easy retrieval.

In the present study, we compared the incidence and fatality rates of colorectal perforation among hospitals in Japan using data in the NCD.

## 2 Methods

### 2.1 Patient selection

**Figure 1:**
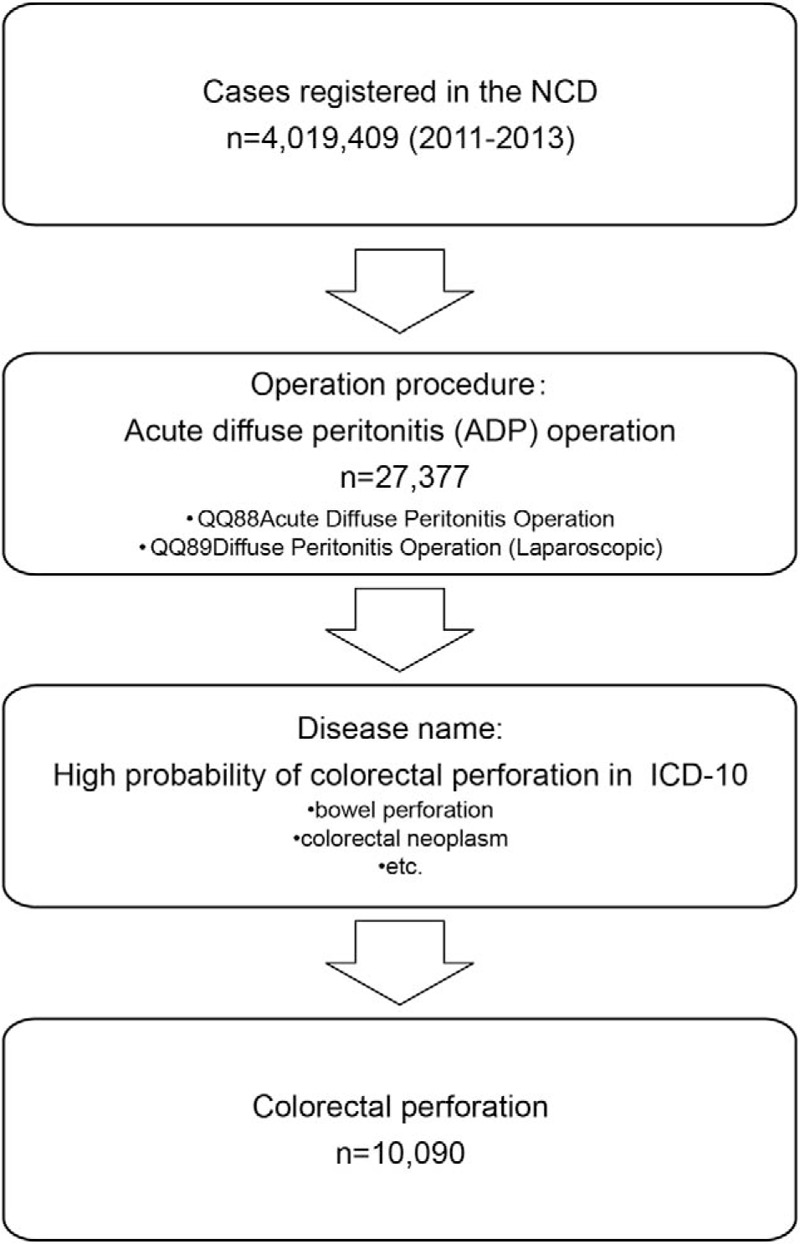
Patient flow chart. We first selected all patients who underwent operations for acute diffuse peritonitis (ADP), because this treatment method is usually used for those with a colorectal perforation. Within this group, we chose patients with a high probability of colorectal perforation, which included those with diseases such as bowel perforation and colorectal neoplasms. NCD = National Clinical Database; ICD-10 = tenth revision of the International Statistical Classification of Diseases and Related Health Problems.

Data from cases registered in the NCD between January 1st, 2011 and December 31st, 2013 were used. All cases of acute diffuse peritonitis (ADP) surgeries recorded in the NCD (QQ88 ADP operation and QQ89 diffuse peritonitis operation [laparoscopic]) were screened, and only patients with a disease that may have caused colorectal perforations were included (Fig. 1). The diseases included bowel perforation, colorectal neoplasms, and other probable conditions listed in the 10th revision of the International Statistical Classification of Diseases and Related Health Problems.

### 2.2 Study design

We categorized Japanese hospitals registered in the NCD into two groups. The collection and use of the data registered in NCD for observational studies were approved by the institutional review boards of the University of Tokyo and Japan Surgical Society. The registry takes an Opt-out approach where the case data are excluded from future analyses upon patients’ request. One group consisted of specialized hospitals that included advanced treatment hospitals,^[9]^ along with emergency and critical care centers.^[10]^ These hospitals were certified by the Ministry of Health, Labour and Welfare (MHLW) and local governments. The other group consisted of non-specialized hospitals.

The 30-day fatality rates and overall surgical fatality rates of the two groups were compared. The primary endpoint measures of this study were the 30-day postsurgery and surgical mortality rates of colorectal perforation (i.e., all patients who died within the index hospitalization period, regardless of the length of hospitalization—up to 90 days).

### 2.3 Advanced treatment hospitals

To be classified as an advanced treatment hospital, a hospital must meet the following criteria: all features of a non-specialized hospital plus an intensive care unit, clean rooms, a pharmaceutical information management system, more than 400 beds, more than 10 departments, and a referral rate of at least 30%. These requirements were established by the MHLW, and they are used to ensure that hospitals can provide high-quality medical care to patients.

### 2.4 Statistical analysis

We used a logistic regression model. Variable selection was used as the forward selection method. Component influences on the mortality rates of colorectal perforation were calculated from the preoperative data (laboratory data, surgical history, and comorbidities) in the NCD using the Fisher exact test, unpaired *t* test, and Mann–Whitney *U* test. Twenty-eight of 178 factors collected from the preoperative variables were used as adjustment factors (Table 1). Using this adjustment model, we compared the observed-to-expected mortality (O/E) ratio to analyze the effect of the two additional factors, with a probability (*P*) value of 0.05 for inclusion. SPSS, version 20 for Windows (IBM Corp., Armonk, NY) was used to perform the data analyses.

**Table 1:**
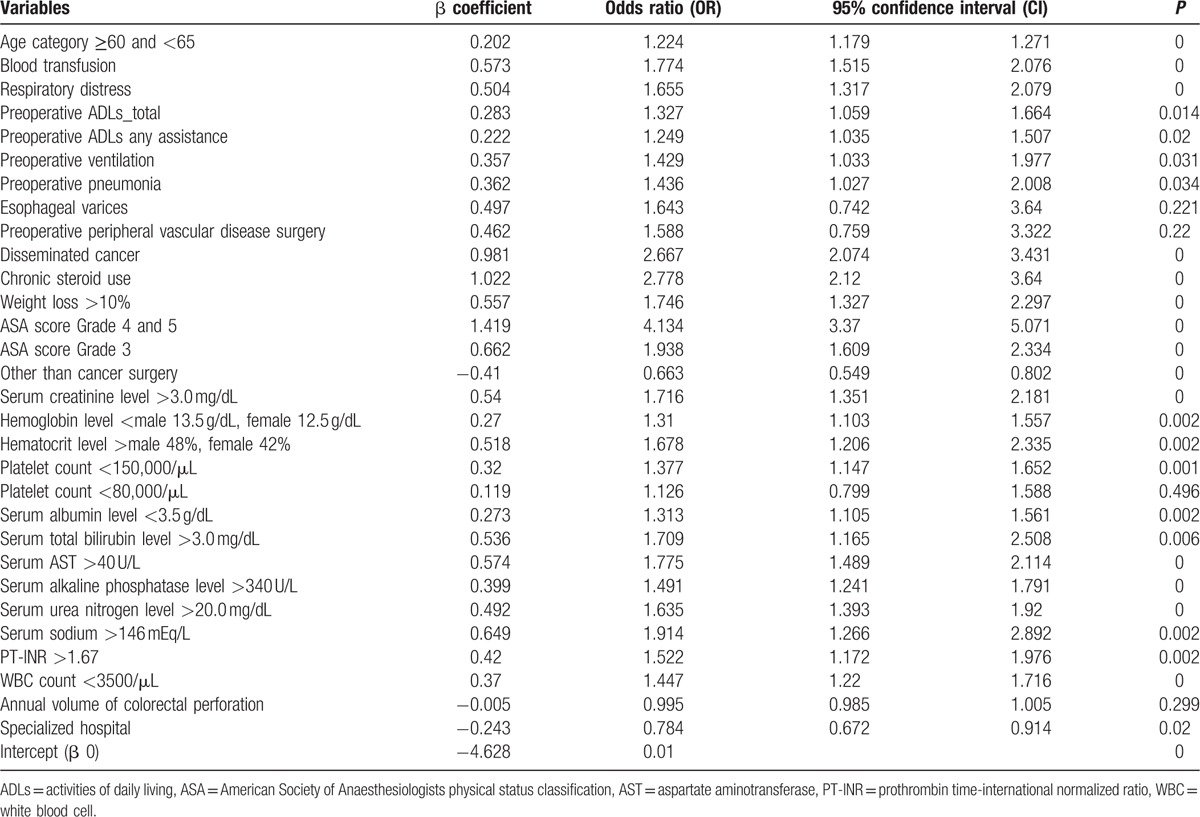
Risk-adjusted factors.

### 2.5 Role of the funding source

This study was supported by a grant from the group of the project study committee for colorectal perforation in the Japanese Society for Abdominal Emergency Medicine.

## 3 Results

### 3.1 Fatality rate of colorectal perforation postoperatively

We used data from 4,019,409 cases registered in the NCD between January 1st, 2011 and December 31st, 2013, from 4181 hospitals. These cases included a total of 27,377 ADP operations. All cases of disease-induced colorectal perforation (10,090) between 2011 and 2013 were included in this study. The overall postoperative fatality rate was 11.36%.

### 3.2 Factors affecting the number of cases of colorectal perforation at each hospital

**Figure 2:**
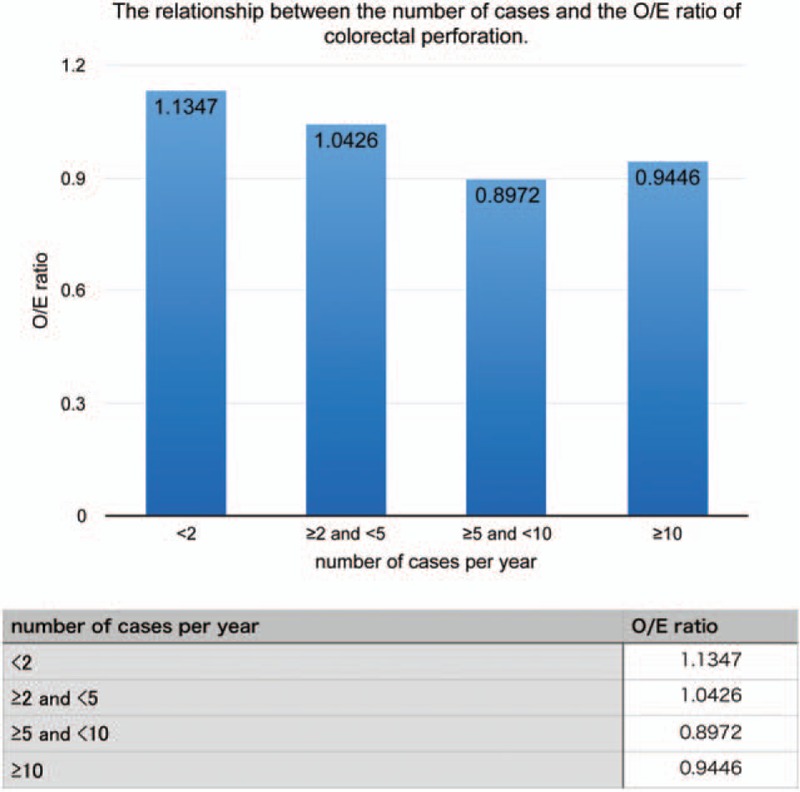
Relationship between the number of cases and the observed-to-expected mortality (O/E) ratio of colorectal perforation. There was a negative correlation between the O/E ratio and the number of cases with colorectal perforation per year. The O/E ratio was slightly higher at hospitals that treated more than 10 patients with colorectal perforation per year due to external factors.

Hospitals that treated more cases per year had lower O/E ratios (Fig. 2), that is, the O/E ratio was lower at hospitals that had more experience with colorectal perforation. The O/E ratio of colorectal perforation was slightly higher at hospitals that had 10 or more cases per year. Nevertheless, the ratio remained low (≤1.0).

### 3.3 Differences between the hospitals

**Figure 3:**
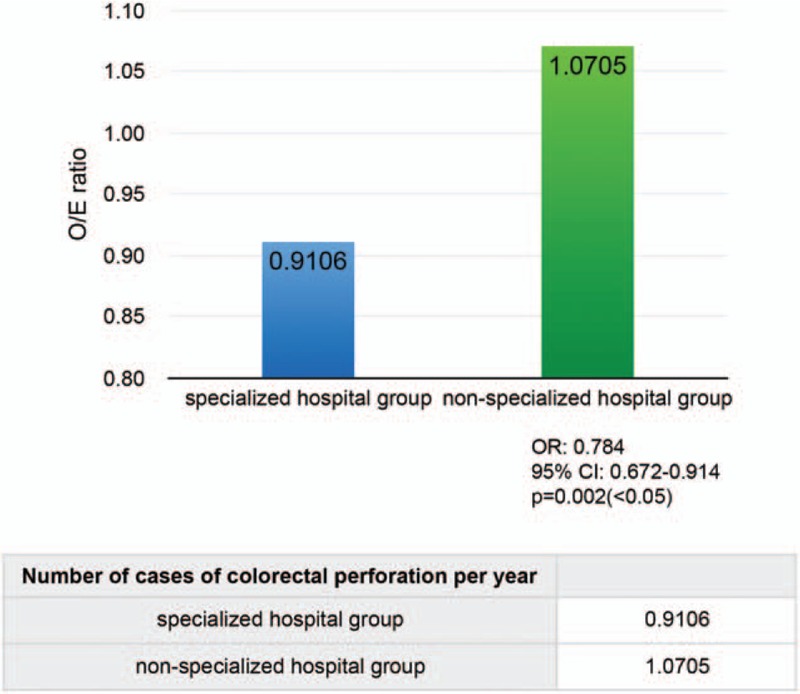
Comparison of the observed-to-expected mortality (O/E) ratio between the specialized and non-specialized hospital groups. There were 3884 patients in the specialized hospital group and 6206 in the non-specialized hospital group. The O/E ratio was significantly lower in the specialized hospital group than in the non-specialized hospital group.

There were 3884 patients in the specialized hospital group and 6206 in the non-specialized hospital group. The O/E ratio was significantly lower in the specialized hospital group (0.9106) than in the non-specialized hospital group (1.0705, Fig. 3) (odds ratio [OR]: 0.784, 95% confidence interval [CI]: 0.672–0.914, *P* = 0.002).

**Figure 4:**
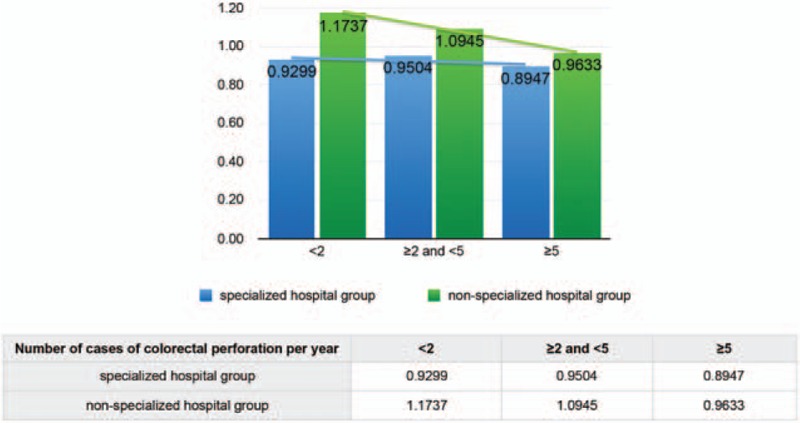
Effect of the number of cases with colorectal perforation per year on the observed-to-expected mortality (O/E) ratio at hospitals registered in the National Clinical Database. A negative correlation was found between the O/E ratio and the number of cases with colorectal perforation per year at both types of hospitals. However, the O/E ratio was significantly lower in the specialized hospital group than in the non-specialized hospital group.

A negative correlation between the O/E ratio and the number of cases with colorectal perforation per year was observed at both hospitals. Figure 4 shows that the number of cases with colorectal perforation per year moderately affected the O/E ratio of the specialized hospital group compared with that of the non-specialized hospital group. However, the E/O ratio was noticeably lower in the specialized hospital group than in the non-specialized hospital group.

### 3.4 The percentage of cases of appendectomy and colostomy

Among all patients in this study, 13.9% (1404 patients) underwent an appendectomy, and 33.5% (3383 patients) underwent colostomy (Table 2). Overall, 27,377 patients who underwent an operation for ADP were recorded in the NCD. Within these patients, a high percentage (33.5%) underwent colostomy. These data suggest that hospitals that treat more cases of colorectal perforation per year were associated with a higher chance of performing colostomies in patients with severe disease. The percentage of colostomies performed increased by more than 10% (from 27.2% to 37.6%) as the number of cases treated by a hospital increased from <2 cases per year to ≥10 cases per year.

**Table 2:**
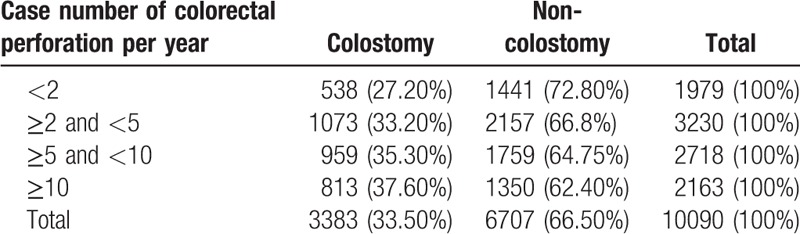
Percentage of patients with colorectal perforation who underwent colostomy.

## 4 Discussion

The O/E ratio of colorectal perforation was 15.98% lower in patients treated at a specialized hospital than the ones treated at a non-specialized hospital. The O/E ratio decreased as the number of cases treated by a hospital increased (Fig. 2). While calculating the O/E ratio of each group, many risk factors (Table 1) were considered and adjustments were made.

The fatality rate was lower in specialized hospitals than in non-specialized hospitals. This result may maybe attributed to the following benefits at the specialized hospitals: (1) there were more doctors in specialized fields; (2) there were more medical staff, which contributed to better care in general; (3) surgeries could be performed at any time of the day; and (4) the number of beds in the intensive care units was higher. In the present study, we observed a negative correlation between the O/E ratio and the number of cases with colorectal perforation per year (Fig. 2). However, the O/E ratio was still slightly higher with 10 or more cases per year than with 5 or more and less than 10. This increase was due to several additional factors. For example, data were included from hospitals that mainly treat severe colorectal perforation (high-risk patients) and from patients who required extra time for hospital transfer. Although the O/E ratio of the non-specialized hospital group was strongly affected by the number of cases with colorectal perforation per year, the effects of the facility were greater than the effect of number of cases with colorectal perforation in the specialized hospital group (Fig. 4). Therefore, the outcome of patients did not depend on the operator's experience in the specialized hospital group.

Based on our results, we suggest that the type of facility was more influential than the number of cases with colorectal perforation in reducing the mortality rate. Yet in Fig. 4, the O/E ratio decreased precipitously with the increase in cases at the non-specialized hospitals, which was similar to the specialized hospitals. Therefore, patients with colorectal perforation should preferably choose to get treated at specialized hospitals or hospitals that treat 5 or more cases of colorectal perforation per year (O/E ratio, 0.9633).

The NCD contains information on 95% of all surgical procedures performed in Japan since 2011. As of the end of 2014, 5,644,957 cases (patients with certain diseases represented as multiple cases) from 4628 hospitals were recorded in the NCD, and the system had 36,088 registered users. The main purpose of this project was to enable surgeons to evaluate the quality of surgical procedures and the outcomes by studying the mortality, morbidity, and distribution of a disease in various regions. All data including variables, definitions, and inclusion criteria for the NCD are accessible online (http://www.ncd.or.jp) to participating hospitals. Risk models for certain procedures such as ADP surgery,^[11,12]^ esophagectomy,^[13]^ total gastrectomy,^[14]^ right hemicolectomy,^[15]^ hepatectomy,^[16]^ and pancreaticoduodenectomy^[17]^ have been created using these data. A risk-adjusted analysis on the basis of NCD data has provided feedback to NCD users on patients’ operative risk.^[18]^ Furthermore, hospitals can be compared using the NCD data, and a summary of the information can be offered to the general public. Thus, the public can identify hospitals most suitable for treating a particular condition. We believe that the NCD is sufficient for establishing that the government policy on healthcare is effective at improving the medical system in Japan.

Because colorectal perforation is not classified as a specific category in the NCD, alternative selection methods were required. First, the category of ADP surgery was selected. Subsequently, several diseases such as bowel perforation and colorectal neoplasms that are commonly associated with colorectal perforations were selected, and patients with these diseases were considered to have colorectal perforations.

Generally, appendicitis is not considered a serious disease, and its fatality rate is low because the appendix is completely removed during treatment. However, there were cases of colorectal perforation associated with appendicitis. Thus, patients who underwent appendectomy were included in the study. About 13.9% (1404 cases) of patients who underwent appendectomy were included.

The current study has several limitations. First, as the category of “colorectal perforation” does not exist in the NCD, other categories (e.g., gastrointestinal perforation and cholecystitis) were used instead to collect relevant data. Additionally, there were no subcategories under ADP operation, which made it difficult to distinguish and isolate all the colorectal perforation-related cases for the study. An update to the NCD with more detailed subcategories is required for future studies.

## 5 Conclusions

We report the first study to examine differences between types of hospitals with respect to the fatality rates of colorectal perforation using the NCD. The O/E ratio was significantly lower in the specialized hospital group (0.9106) than in the non-specialized hospital group (1.0704), specifically 15.98% lower. Hospitals can be certified as a specialized hospital only if they strictly follow all the requirements and regulations set by the government. The results of this study indicate that specialized hospitals may provide higher quality medical care, which in turn proves that government policy on healthcare is effective at improving the medical system in Japan.

## Acknowledgments

The authors thank all the data managers and hospitals for participating in the NCD project and for their great effort in entering the data. They also thank Chieko Fujimura and Hitomi Okamoto.
