# A comparison of the surgical mortality due to colorectal perforation at different hospitals with data from 10,090 cases in the Japanese National Clinical Database: Erratum

**DOI:** 10.1097/MD.0000000000006956

**Published:** 2017-05-19

In the article, “A comparison of the surgical mortality due to colorectal perforation at different hospitals with data from 10,090 cases in the Japanese National Clinical Database”,^[1]^ which appeared in Volume 96, Issue 2 of *Medicine*, Dr. Koichi Hirata's affiliations appeared as affiliations j and k, but should have appeared as j, k and l.
